# The French Cohort of DNA Repair-Deficient Xeroderma Pigmentosum Patients: Risk of Hematological Malignancies

**DOI:** 10.3390/cancers15102706

**Published:** 2023-05-10

**Authors:** Alain Sarasin

**Affiliations:** 1 Unité Mixte de Recherche UMR9019 Centre National de la Recherche Scientifique, 94805 Villejuif, France; alain.sarasin@gustaveroussy.fr; 2 Gustave Roussy Institute, 94805 Villejuif, France; 3 Université Paris-Saclay, 91400 Saclay, France

**Keywords:** nucleotide excision repair, xeroderma pigmentosum, UV light, skin cancers, internal tumors, leukemia

## Abstract

**Simple Summary:**

Xeroderma pigmentosum (XP) is a genetic disease caused by DNA repair deficiency. We diagnosed 181 XP patients from 1982 to 2022 in France. The patients are very sensitive to sunlight and will rapidly develop skin cancers in exposed body sites. However, thanks to better protection, XP patients live much longer than in the past. This better survival is associated, for the French XP patients originating from North Africa and bearing the same founder mutation in the *XPC* gene, with a very high risk to develop aggressive and lethal internal cancers, particularly hematological malignancies, and brain, gynecological, and thyroid tumors. This is a new threat hanging over all XP-C patients. The molecular origins of internal tumors in this particular set of DNA repair-deficient patients are discussed. It is important that physicians and XP families be aware of this high risk to prevent and look for early diagnosis of internal tumor development in XP patients.

**Abstract:**

Background: Xeroderma pigmentosum (XP) is a rare genetic disorder characterized by a high incidence of skin cancers. These patients are deficient in nucleotide excision repair caused by mutations in one of the 7 XP genes. Methods: We diagnosed 181 XP patients using UV-induced DNA repair measurements and/or DNA sequencing from 1982 to 2022 in France. Results: As all XP patients, the French ones are very sensitive to UV exposure but since they are usually very well protected, they develop relatively few skin cancers. A majority of French XP patients originate from North Africa and bear a founder mutation on the *XPC* gene. The striking discovery is that these patients are at a very high risk to develop aggressive and lethal internal tumors such as hematological malignancies (more than a 100-fold risk compared to the general population for myelodysplasia/leukemia) with a median age of death of 25 years, and brain, gynecological, and thyroid tumors with even lower median ages of death. The high mutation rates found in XP-C internal tumors allow us to think that these XP patients could be successfully treated by immunotherapies. A full analysis of the molecular origins of these DNA repair-deficient tumors is discussed. Several explanations for this high predisposition risk are proposed. Conclusions: As the age of the XP population is increasing due to better photo-protection, the risk of lethal internal tumors is a new Damocles sword that hangs over XP-C patients. This review of the French cohort is of particular importance for alerting physicians and families to the prevention and early detection of aggressive internal tumors in XP patients.

## 1. Introduction

Xeroderma pigmentosum (XP) is a rare, genetic, recessive disease, due to a deficiency in nucleotide excision repair (NER) [1,2]. Symptoms of XP include photosensitivity, actinic keratosis, and early ocular injuries. Early onset of cutaneous tumors on UV-exposed skin occurs with a thousand times higher frequency than in the general population [3,4,5]. Seven complementation groups are involved in the classical XP disease, caused by germline mutations in one of the *XPA*, *B*, *C*, *D*, *E*, *F*, or *G* genes [2,6]. The XP Variant (XP-V) form is NER proficient but is caused by mutations in the *POLH* gene coding for the translesion synthesis DNA polymerase η [7,8]. XP is a rare disease with an incidence of 1–2/1,000,000 in Europe [9] and USA [10]. It is more common in some isolated countries such as Japan 1–5/100,000 [11] and Comorian Islands 1/10,000 [5]. One of the highest incidences of XP (>1/50,000) occurs in Tunisia, Algeria, and Morocco (called later North Africa) and is associated with a high allelic frequency of a founder *XPC* mutation in the population with traditions of consanguinity [12].

The main DNA lesions repaired by NER are the UV-induced CPD (cyclobutane pyrimidine dimers) and 6-4PP ((6-4) pyrimidine–pyrimidone adducts) [13], but NER is also able to remove various bulky lesions such as those induced by genotoxins contained in cigarette smoke, genotoxic food contaminants, for example, the hepato-carcinogen Aflatoxin B1 [14], or some ROS-induced DNA damage [15]. 

Before the 1980s, XP patients in France were only clinically diagnosed by dermatologists according to their sun sensitivity, their skin disorders, and the early appearance of skin tumors. As the author had worked with cultured XP cells in the laboratory of Prof. P.C. Hanawalt at Stanford University (USA) [14], some French dermatologists asked me to help them with a biological and molecular XP diagnosis as well as developing a prenatal diagnosis of DNA repair-deficient diseases [16]. The author’s laboratory developed the cell cultures of XP from skin biopsies, the measure of nucleotide excision repair by unscheduled DNA synthesis, and the complementation group determinations. At the beginning of the 1980s, my laboratory was fully efficient for diagnosing and characterizing the different XP patients, including the variant form, as it was also available in some other countries [15]. We constructed and characterized the first SV40-transformed XP-C cell line that was used worldwide [17]. Registration of the French XP cohort started, therefore, around 1982. 

We report here our experience of diagnosing from 1982 to 2022, by molecular analysis, most of the XP patients living in France. We describe this French cohort of 181 patients in terms of complementation groups, genetics, and internal tumor appearance. The major purpose of this review is to stress the high risk of developing aggressive and lethal internal tumors in some specific classes of French XP-C patients and propose a better follow-up of these patients by their physicians.

## 2. Materials and Methods

### 2.1. French Cohort

We have diagnosed DNA repair-deficient diseases for 40 years from 1982 to 2022. Among 181 diagnosed XP patients during that period, we reported 36 internal tumors [4,18,19,20,21,22,23,24], and moreover, we recently identified 7 new tumors, all of which are listed in Table 1. All patients were seen and treated in university hospital centers in France and sometimes in North Africa. Skin biopsies and blood samples were received in the Laboratory of DNA repair-deficient diseases at the Gustave Roussy Institute (Villejuif, France) or the Hematology Center at Saint-Louis Hospital (Paris, France) for molecular diagnosis. DNA repair activities, determination of XP complementation groups, and Sanger sequencing of XP genes were performed as already published [5,16,25,26,27]. 

### 2.2. Statistical Analysis

Kaplan–Meier estimates were used to compare survival between different patient groups (https://biostatgv.sentiweb.fr/?module=tests/surv; accessed on 15 February 2023). Results with *p* < 0.05 were considered significant. The distribution and incidence of cancer patients in the French general population are available as “National estimates of cancer incidence and mortality in metropolitan France between 1990 and 2018”; Vol. 2, *Hematological Malignancies* (https://www.e-cancer.fr/; accessed on 18 January 2023) [28].

## 3. Results

### 3.1. The French Cohort of Xeroderma Pigmentosum Patients

**Figure 1:**
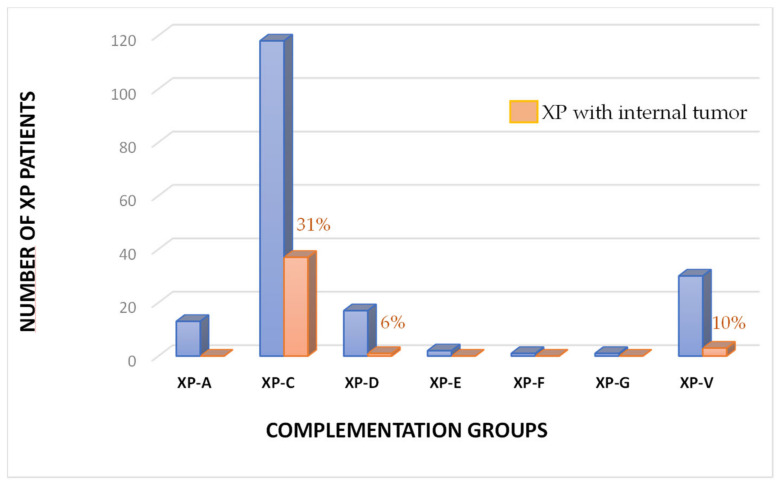
Percentage of XP patients with internal tumors in each complementation group among the French XP patients. The blue bars represent the true number of XP patients and the orange bars are the percentage of internal tumors for each of the 7 complementation groups available among the French XP patients.

These XP patients were sent to us by dermatologists who diagnosed them clinically according to their sensitivity and their skin modifications following minor sun exposure. Between 1982 and 2022, we diagnosed 181 XP patients on UV-irradiated skin biopsies by Unscheduled DNA Synthesis (UDS) followed by complementation analysis and/or DNA sequencing. The complete description of these patients as well as their clinical information are given in Supplementary Table S1. Among them, 13 patients belong to the XP-A group, 117 to the XP-C, 17 to the XP-D, 2 to the XP-E, 1 to the XP-F, 1 to the XP-G, and 30 to the XP-V group (Figure 1). As expected for an autosomal recessive disease, the ratio of men to women is 1.04. 

In addition to patients of French origins (20%), the majority (66%) of XP patients living in France belong to families originating from North Africa. Among the 68 known *XPC* mutations causing XP, almost all of these Maghrebi XP-C patients in our cohort bear the founder *XPC* homozygous mutation: c.1643_1644 delTG; p.Val548AlafsX572 (called later delTG), two patients have heterozygotes delTG mutation and two patients have a different *XPC* mutation (Supplementary Table S1). We, previously, estimated that this mutation appeared around 1250 years ago in North Africa, probably coming from the Middle East, indicating that all these patients should have common ancestors [27]. These patients with the delTG mutation are, like all XP-C ones, very sensitive to UV exposure, without neurological abnormalities but, as we discovered later, are at high risk of developing internal cancers. 

### 3.2. Skin Cancers in the French XP Cohort

**Figure 2:**
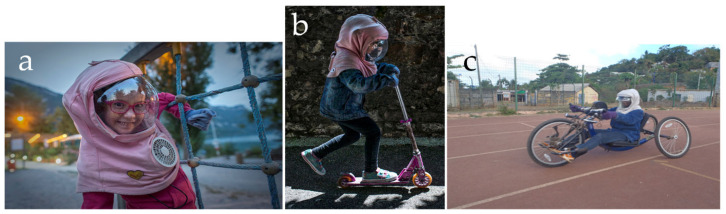
Full face protection of XP patients. Full ultraviolet protections of the face of XP patients have been constructed allowing them to be outside on a sunny day without any danger and without any sunlight-protective cream. The XP patients can participate in a normal activity with this protection (**a**), such as skating (**b**) or cycling, at noon time, on the Comorian Island Mayotte very close to the equator (**c**). Photographs are shown with the agreement of the XP patients and their families. Thanks to Fabrice Dimier for shooting the (**a**,**b**) pictures.

Like all XP patients, the French ones are very sensitive to sun exposure but this sensitivity varies according to the complementation groups. The XP-C group is the most sensitive one with the very early appearance of skin abnormalities following short sun exposure and early development of skin cancers if exposed to sunlight. Thanks to the French XP support group, called *Association des Enfants de la Lune*, full UV-protection clothing has been developed with an astronaut-type of face protection, which is freely given to newly diagnosed XP children in France (Figure 2a). This face protection is associated with some type of air conditioning allowing XP teenagers to do sport even in tropical areas without any difficulties (Figure 2b,c). The positive result of this protection is that French XPs almost do not develop skin cancers anymore. We have adult XPs (more than 25 years old) without any skin cancers and we believe that their life expectancy will tend toward the normal one. Moreover, an international study on the ability of XP patients to adhere to photoprotection showed that the French patients were, indeed, very adherent to face and body photoprotection [29]. This is a clear confirmation of the major role of sun exposure in the induction of skin cancers.

### 3.3. Internal Tumors in the French XP Cohort 

As the age of the XP population is increasing due to better sun protection and because all the cells of an XP patient are deficient in DNA repair, one can hypothesize that these individuals may develop internal tumors while aging. In the French XP cohort, a series of 43 internal tumors are described in Table 1 corresponding to 40 XP patients (3 patients had 2 unrelated tumors). Over 40 years, we have listed 23 hematological malignancies (53%), 8 gynecological tumors (19%), 3 brain tumors (7%), and 3 thyroid tumors (7%). Only one patient has been found for either the stomach, prostate, pancreas, breast, or kidney sites as well as one angiosarcoma (Table 2). The vast majority of the XP patients (90%) with internal tumors belong to the XP-C group (Figure 1). Among them, 95% exhibit the delTG founder mutation (Table 1). If 31% of the XP-C patients develop internal tumors, this number is only 10% for XP-V at later ages and 6% for XP-D patients (Figure 1, Table 2). In XP-A patients we did not report internal tumors, which might be associated with a small life expectancy due to the very strong neurological deterioration. 

Among the 23 hematological malignancies, 10 patients have been reported with a general diagnosis of leukemia but at least 8 patients had Acute Myeloid Leukemia (AML). Among them, 2 XPs are still alive (April 2023) including 1 patient following Hematopoeitic Stem Cell Transplant (HSCT). Three patients died of Myelodysplasia (MDS) and five patients had MDS that transformed into AML (including the only living one waiting for HSCT), three patients died of lymphoma, three patients had Acute Lymphoblastic Leukemia (ALL) (two ALL which developed later into a clonal expansion of MDS and/or AML-6), and one XP-D patient died of Hodgkin’s disease following chemotherapy (Table 1). 

**Figure 3:**
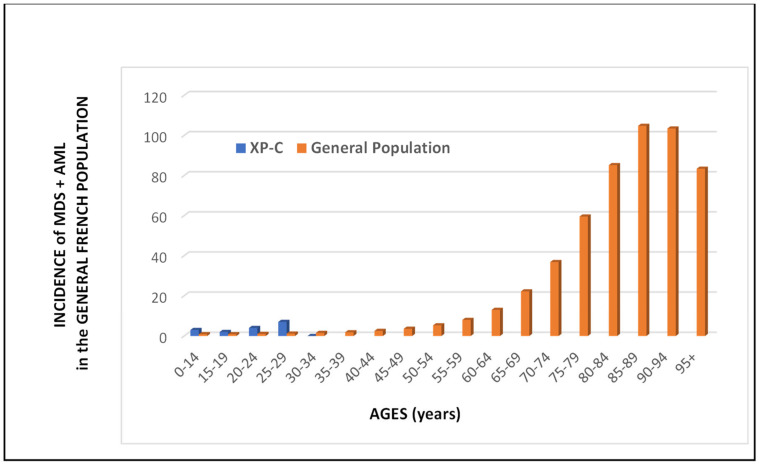
Incidence of combined myelodysplasia (MDS) and Acute Myeloid Leukemia (AML) in the French general population and number of XP-C patients with MDS and/or AML. In orange, incidence of MDS and AML in the French population according to the ages for 100,000 person-years (data have been compiled in 2018 and are available as “National estimates of cancer incidence and mortality in metropolitan France between 1990 and 2018”; Vol. 2, *Hematological Malignancies*; https://www.e-cancer.fr/; accessed on 18 January 2023 [28]). In blue, the true number of XP-C patients with MDS and/or AML: 3 patients for ages 0–14, 2 for 15–19, 4 for 20–24, and 7 for 25–29 (see Table 1).

**Figure 4:**
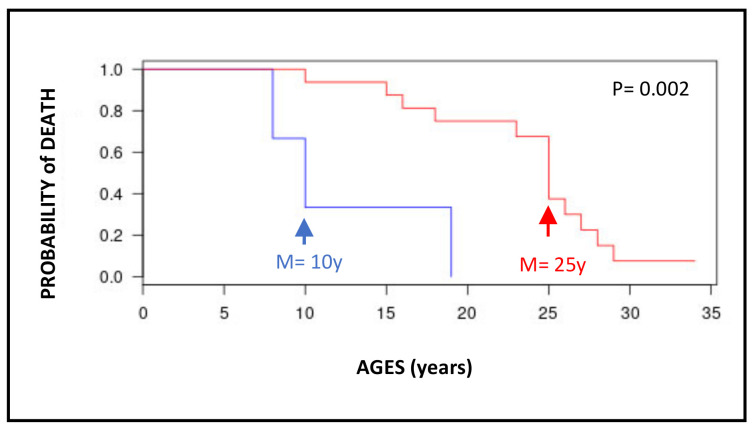
Kaplan–Meier distribution of survival for XP-C patients with brain tumors (blue curve) or MDS/AML (red curve). Three XP-C patients developed early brain tumors (median ages of 10y) and 16 XP-C patients developed MDS and/or AML (median ages of 25y). The two curves are statistically different (*p* = 0.002; X^2^ test).

The median ages at tumor diagnosis are very young, especially for the brain (10y), thyroid (17y), and gynecological (17y) tumors (Table 2). For the hematological malignancies, if one analyzes only MDS and AML, the median age at diagnosis is 25 years for XP-C patients while it is 79 and 71 years, respectively, for the French general population (Figure 3). It is difficult to really compare the incidences of MDS/AML between the French XP patients and the French general population because of the very low number of XP patients. However, the disparities between the two curves are extremely obvious by looking at Figure 3. In the general population, MDS/AML are really diseases of aged people while all MDS/AML XP-C patients are less than 30 years old. The patient survival after the diagnosis is relatively short, less than 2 years, for brain tumors and hematological malignancies (Table 1). However, the survival of XP-C patients with MDS/AML is statistically better than that of XP-C with brain tumors (Figure 4). The gastric and prostate tumors occurred in middle-aged XP-V patients (around 50–60y) which may correspond to the usual ages in the general population (Table 2).

### 3.4. Cautious Use of Chemotherapy for Treating Cancers in XP-C Patients

Internal tumors are usually treated by chemotherapy in the general population. Most chemotherapeutic drugs damage DNA in order to kill the actively replicating tumoral cells. For example, cisplatin and its derivatives induce intra- and inter-strand DNA crosslinks leading to DNA replication, transcription, and cell cycle arrests. The lack of nucleotide excision repair of the lesions induced by these drugs in XP patients could render, in theory, more efficient the anti-tumoral treatment [30]. Unfortunately, chemotherapy in XP patients is so efficient that it can kill too many normal cells and be toxic for the patient. In fact, in our French cohort, seven patients died following chemotherapy. This does not imply that the patients would have survived the disease without treatment, of course, but this clearly indicates that before starting a chemotherapeutic protocol in an XP patient, one must be aware of the toxicity risk. In addition to the French cohort, several XP patients have been reported to die from adverse effects attributed to cisplatin treatment [31,32] leading to myelosuppression and multiple organ failure. Indeed, several in vitro reports show increased cytotoxic effects of cisplatin or doxorubicin on classical XP cells [30,33]. If chemotherapy appears to be necessary, one can recommend using specific drugs that do not damage DNA, if possible, and if not, to considerably reduce the utilized doses. Due to the low survival of XP patients with AML, allogeneic hematopoeitic stem cell transplants are often proposed. However, the usual conditioning regimen with efficient DNA-damaging agents is also very toxic for XP patients [34]. The XP2006VI French patient received a reduced intensity conditioning regimen, analog to that given to Fanconi’s Anemia patients who are also very sensitive to chemotherapy [20]. The XP2006VI patient is still in complete response of her leukemia (April 2023).

It is usually admitted that cancers with a high tumor mutation burden have a good response to Immune Checkpoint Inhibitors (ICIs such as Pembrolizumab, Ipilimumab, or Nivolumab). Since XP tumors show a high mutation level [21,35,36], one can hypothesize that some XP tumors could be sensitive to these drugs. Indeed, the patient XPWaVI (33 years old) has been treated for his angiosarcoma of the inner canthus of the left eye with 34 cycles of Nivolumab (3 mg/kg every 2 weeks) reaching complete remission within 5 months [23]. Similarly, patient XP819VI was successfully treated with 27 cycles of Pembrolizumab for a liver metastasis.

## 4. Discussion

XP patients are characterized by a dramatic hypersensitivity to minimal sun exposure, caused by a deficiency in NER producing a high level of somatic mutations [18,21,35,36] and inducing numerous skin cancers at early ages with a thousand times higher frequency than in the general population [1,2,3,4,5]. NER is also involved in the repair of other bulky DNA lesions than UV-induced DNA damage [14,15,37,38]. As XP patients live longer now and because they exhibit a systemic defective nucleotide excision DNA repair process, an increased mutation rate and associated cancer risk in tissues not exposed to sunlight may be expected. By reviewing the French XP cohort over a 40 years, it is striking to count a large number of internal tumors in patients of young ages and particularly hematological malignancies. We already reported that the odds ratio for the risk to develop hematological malignancies was more than 100 in XP patients as compared to the general population [22]. This risk factor was higher for the French XP compared to the world XP population and particularly for those bearing the North African delTG mutation. Indeed, in our cohort, all hematological malignancies of XP-C patients are found in delTG XP-C patients (Table 2).

**Figure 5:**
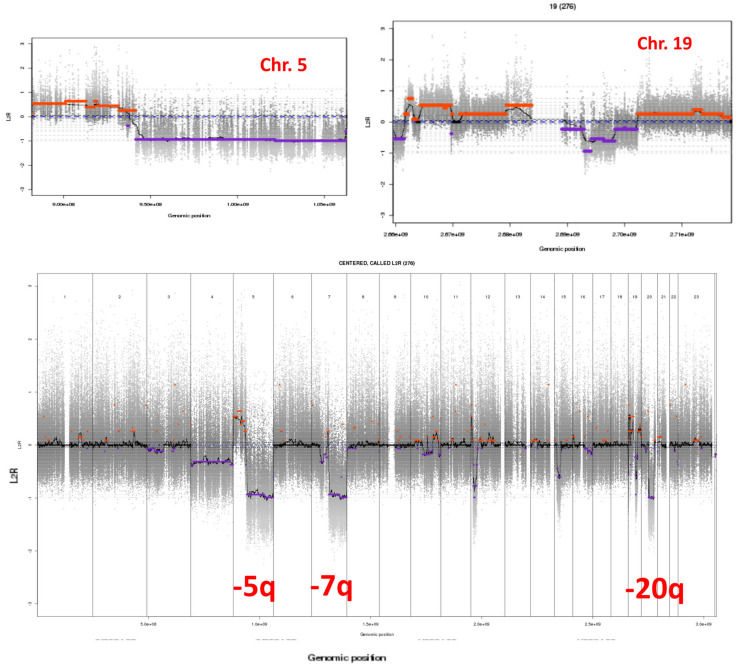
Somatic chromosomal abnormalities in the AML with myelodysplasia-related changes in the XP185VI patient. Patient XP185VI first developed MDS (at 24 years old) followed by AML-6 at 25y. One can see numerous chromosomal changes, especially in chromosomes 5q and 7q. Below the centered-line one can see loss of genomic materials in blue and above the centered-line one can see gain of genomic materials in red.

The most striking characteristics of XP-C hematological malignancies are that they resemble MDS/AML observed in the general population of patients previously treated by chemotherapy for a first cancer [20]. For example, *TP53* mutations and complex karyotypes are found in all secondary MDS/AML as also observed in our XP-C patients with the delTG mutation [18]. As an example, cytogenetic analyses of blast cells isolated from the bone marrow of the XP185VI patient, who developed MDS transformed into AML-6, clearly show somatic chromosomal abnormalities in the AML with myelodysplasia-related changes such as 5q, 7q, and 20q deletions and numerous alterations on chromosome 19 (Figure 5). Del5q and del7q complex karyotypes are often associated with therapy-related MDS/AML, but of course, the young XP patients were not treated by any anti-tumoral protocol before diagnosis. This similarity strongly suggests that young XP patients rapidly accumulated spontaneous DNA lesions in their internal cells (similar to cancer patients accumulating DNA lesions following treatment by chemotherapy) that were not repaired due to NER deficiency and therefore giving rise to numerous mutations and tumoral processes.

NER is composed of two sub-pathways: the transcription-coupled repair (TCR) able to specifically repair bulky DNA lesions on the transcribed strands of active genes and the global genome repair (GGR) able to repair DNA lesions all over the genome except on transcribed strands [1,2,6,15,39]. XP-C and XP-E are proficient in TCR but deficient in GGR, while the other complementation groups are deficient in both TCR and GGR; XP-V is NER proficient [6,7,8]. When TCR- and GGR-deficient cells, such as XP-A or XP-D patients, experience DNA damage, DNA replication, transcription, and cell cycle are unscheduled-blocked because of the total absence of lesion removal. This cell cycle blockade and the arrest of RNA polymerase II-driven transcription induce P53-dependent apoptosis at low UV doses in TCR/GGR-deficient XP cells [40,41,42,43]. This implies that damaged XP-A or XP-D cells will undergo apoptosis following DNA-damaging treatment and the dead cells will obviously not give rise to mutations and cancers. Indeed, these patients develop much fewer skin cancers and internal tumors than TCR-proficient XP-C and XP-E patients [3] (Figure 1). In contrast, TCR-proficient cells are able to remove DNA lesions located on the transcribed strands allowing, therefore, normal transcription and only partial blockade of the cell cycle. Replication in XP-C or XP-E cells in the presence of unrepaired DNA lesions on non-transcribed strands will lead to mutations, probably using translesion synthesis DNA polymerases, and partially blocked DNA replication forks will be overcome by using several recombination pathways. The final result is the production of point mutations and chromosomal abnormalities as observed in XP-C AML (Figure 5). This explains why XP-C and XP-E patients (but XP-E patients are very rare) are more likely to have early onset of skin cancers than XP patients who are TCR and GGR deficient (XP-A, XP-B, XP-D, and XP-G) [3,35]. This is also true for internal cancers because in the French cohort, 31% of XP-C patients developed internal tumors while it was only 6% for XP-D patients and none for XP-A patients (Figure 1).

**Figure 6:**
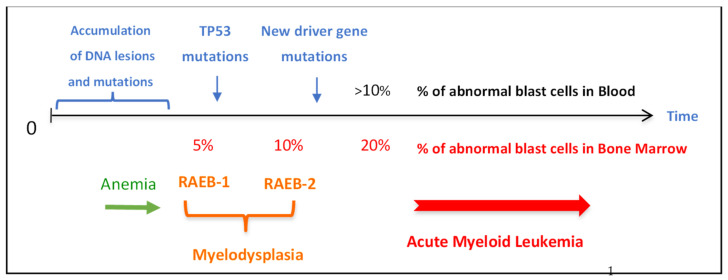
Timing of the appearance of major hematological abnormalities in French XP patients. Often XP patients develop anemia before the appearance of MDS (RAEB-1 and -2 correspond to the old name for MDS, meaning Refractory Anemia with Excess Blasts) which occurs with a median age of around 24 years old. MDS are rapidly transformed into AML. Numerous driver gene mutations and chromosomal abnormalities are associated with these hematological malignancies.

We reported a 25-fold higher mutation level in XP-C hematological malignancies than in the same tumor types in the general population with a very strong bias toward mutations caused by lesions located on non-transcribed strands [21]. The mutation analysis showed an increase of G to T and A to T base substitutions in XP-C hematological malignancies as compared to sporadic ones. The mutated purines were all located on the untranscribed strands of active genes [21]. Interestingly enough, it has been reported in 1-year-old NER-deficient *Xpc^-/-^* mice a 30-fold increase in spontaneous mutant frequency in T-lymphocytes. The mutation spectrum is essentially GC to TA transversions in which all affected Gs are exclusively located in the non-transcribed strand [44]. This increase did not happen in old *Xpa^-/-^* or *Csb^-/-^* TCR-deficient mice probably because the damaged cells are eliminated by apoptosis [44]. The somatic mutations found in the studied XP-C hematological malignancies closely resemble COSMIC signature 8 [21,45], indicating the induction of unrepaired spontaneous purine DNA lesions probably produced by an endogenous oxidative process [15,18,21,37,38,46,47]. Several purine lesions produced by oxidative stress are fully repaired by NER (such as purine cyclodeoxynucleotides) [38,46] or partially need NER (such as 8-oxo-dG) [47]. These lesions that are probably induced in all organisms during normal life and aging should be repaired by NER but in XP patients, these unrepaired DNA lesions will rapidly accumulate with time leading to mutations and early cancer development. As shown in Figure 6, one can hypothesize that DNA lesions accumulate all over life starting in infancy in all individuals but due to the lack of NER, the XP-C cells will accumulate mutations and particularly in organs that constantly replicate, such as the hematopoietic system. MDS can appear in patients as early as 15 to 20 years of age with blasts only detected in the bone marrow. Then, this malignancy can be transformed into AML with the appearance of blast cells in the blood around the age of 20 to 25 years. Interestingly, some French XPs had anemia several years before the appearance of MDS [20]. This could be an easy marker for the early detection of hematological disease in XP patients (Figure 6).

Predisposition to central nervous system tumors has been reported in the American and English XP cohorts [48,49] and thyroid nodules in the American XP cohort [50]. However, the very high predisposition to hematological malignancies only observed for French XP-C patients should be linked to the genetics and the consanguinity of this North African population with the delTG mutation. Besides leukemia also found in XP-C patients living in North Africa [12], three XP patients carrying the delTG mutation and hematological malignancies have been described in the USA and one XP patient with delTG had ovarian cancer in Brazil [51,52]. WGS of various families originated from North Africa with XP patients carrying the delTG germline mutation and leukemia did not show any abnormalities, except the run of homozygosity at the level of the *XPC* gene (chromosome 3p25.1), and did not allow us to propose any identified genetic cause for this leukemia predisposition [20,21]. The delTG mutation gives rise to a stop codon and a short XPC protein may be produced but is undetectable by Western blot analysis using several anti-XPC antibodies [27,53]. This is not exceptional because more than 90% of *XPC* mutations described around the world produce a stop codon with a relatively normal level of mRNA and short unstable proteins. Although we cannot characterize a clear genetic cause for internal tumor development in delTG patients, it is striking to report two sets of North African delTG cousins with AML and MDS and two sisters with gynecological tumors (Table 1). Moreover, we described an Algerian family with XP-C delTG homozygotic twins (XP2003VI and XP2004VI) who developed, at the same age (16 years old), vaginal embryonal rhabdomyosarcoma (Table 1). These tumors were successfully treated by radio- and chemo-therapies but unfortunately, patient XP2004VI died 7 years later following AML which was probably induced by the therapy [21].

The way of living of the delTG patients with North African traditions and common food may be also specific to this population. One can hypothesize that specific food contaminants or additives may exacerbate potential oxidative internal DNA damage. Some uncharacterized food contaminants may be present specifically in North African food, such as Acrolein, linked to the way of cooking or the genotoxin Aflatoxin B1 that leads to exocyclic mutagenic dG damage [14,54]. We demonstrated that the carcinogen Aflatoxin B1 induced guanine DNA lesions that are repaired by NER [14] and that XP cells in vitro were very sensitive to it. Aflatoxin B1 is present in the food in Sub-Saharan Africa and induces liver cancers. Similarly, other types of genotoxic contaminants might be present in some common food in the North African population and be particularly carcinogenic in XP-C patients. It is known that some pesticides, fungicides, and herbicides are producing reactive oxygen species that can damage cellular organelles and DNA. For example, the herbicide Paraquat has been shown to produce chromosome abnormalities, DNA breaks, and micronuclei in treated mice [55]. These DNA damage should be more toxic in an XP-background. One can, therefore, imagine that the high predisposition to internal cancers in XP-C delTG patients is caused by a combination of genetic anomalies and environmental factors specifically linked to this particular population.

## 5. Conclusions

The XP patients of the French cohort are characterized by a very high risk to develop internal tumors such as hematological malignancies, and gynecological, brain, and thyroid cancers. The very high predisposition to leukemia at early ages is associated with a common haplotype and the founder North African delTG mutation as well as probably other factors linked to the Maghrebi population. As French XP patients are better protected and live longer now than in the past, we may anticipate seeing more and more internal tumors in this population. 

The medical doctors who follow XP patients must be aware of this strong predisposition. Early detection of thyroid and gynecological cancers is easy to develop regularly in these patients. For hematological malignancies, blood analysis can be performed every year. For example, often the MDS/AML occurring in XP-C patients appeared following several years of anemia [20], which should be searched for by a regular blood analysis every year starting around the age of 10.

Dissecting cancer occurrence in XP patients can also help us to better understand and prevent tumor development in the general population. These data open a new insight into the role of NER on DNA lesions spontaneously induced by an endogenous oxidative process.

## Figures and Tables

**Table 1:** Clinical and genetic description of the internal tumors developed by the French XP patients.

Patient’s	Countries of	Sex	Ages	Ages at	XP	XP Gene	Clinical Information	References
Code	Family’s Origin		In 2023 (Years)	Death (Years)	Group	Mutations		
XP10VI *	Morocco	M		28	XP-C	delTG	AML-4 (27y); death after chemotherapy at 28.	[20]
XP82VI *	Tunisia	M		18	XP-C	delTG	AML-6 (16y); HSCT (17y); death of toxicity.	[20]
XPAlHaVI	Tunisia	M		25	XP-C	delTG	Death of AML.	[20]
XP128VI	North Africa	F		16	XP-C	delTG	Leukemia (15y); death following chemotherapy.	This paper
XP148VI	Algeria	F		19	XP-C	del TG	Poorly differentiated follicular thyroid carcinoma (13y).	[18]
XP155VI	USA	M		42	XP-V	?	Death of T-lymphoma following chemotherapy.	This paper
XP165VI	Morocco	F		25	XP-C	delTG	Kidney adenocarcinoma (23y).	[4]
XP167VI	Algeria	M		26	XP-C	delTG	Trisomy 21; MDS (25y); death of AML.	[20]
XP185VI	Spain	F		25	XP-C	delTG	MDS (24y); death of AML.	[20]
XP208VI	Algeria	M		17	XP-C	delTG	Mediastinal lymphoma (8y).	[22]
XP233VI	Tunisia	M		8	XP-C	delTG	Death of anaplastic astrocytoma.	[19]
XP235VI	Tunisia	F		29	XP-C	delTG	MDS (24); death of AML-6.	[20]
XP269VI	Morocco	F		23	XP-C	delTG	Cervical tumor (18y).	[4]
XP309VI	Morocco	F		10	XP-C	delTG	B-ALL (7y); death of MDS following chemotherapy.	[20]
XP420VI	Morocco	F		25	XP-C	delTG	Death of cutaneous lymphoma NK.	[22]
XP538VI	Algeria	M		29	XP-C	delTG	Death of AML-2.	[20]
XP664VI	Tunisia	M		10	XP-C	delTG	Death of astrocytoma.	This paper
XPAdSaVI	Morocco	M		19	XP-C	delTG	Cerebellar astrocytoma (14y).	[22]
XP673VI	Morocco	F		22	XP-C	delTG	Death of T-ALL.	[20]
XP694VI	North Africa	F	21		XP-C	delTG	Left ovarian juvenile granulosa-cell tumor at 19y	[24]
							and right ovarian Sertoli–Leydig cell tumor at 19y.	
XP757VI	French	M		20	XP-D	?	Hodgkin’s disease (20y); death following chemotherapy.	This paper
AS802VI	Algeria	M	33		XP-C	delTG	Poorly differentiated follicular thyroid carcinoma (20y).	[4]
XP819VI	French	M	73		XP-V	c.1727_1728delCT; c.883G>A	Prostate tumor at 60y.	[22]
XP820VI	North Africa	F	19		XP-C	delTG	MDS/AML in January 2023; waiting for HSCT.	This paper
XP924VI	Morocco	M		15	XP-C	delTG	T-ALL (12y); MDS (13y); death of AML-6.	[4]
XP2003VI **	Algeria	F	28		XP-C	delTG	Vaginal embryonal rhabdomyosarcoma at 16y.	[22]
XP2004VI **	Algeria	F		23	XP-C	delTG	Vaginal rhabdomyosarcoma at 16y and death of AML.	[22]
XP2006VI	Morocco	F	34		XP-C	delTG	AML-6 (29y); HSCT (29y).	[20]
XPGAVI	French	F		54	XP-V	?	Gastric tumor (48y).	[17]
XPAHVI	Tunisia	M		25	XP-C	delTG	AML (24y); curietherapy (24y); death at 25.	[20]
XPChFa2VI	North Africa	M		24	XP-C	?	Death of leukemia.	This paper
XP2020VI	Tunisia	F	14		XP-C	delTG; c.G850T	Ovarian Sertoli–Leydig cell tumor (11y).	[24]
XPAAVI	Algeria	F	29		XP-C	delTG	Papillary thyroid carcinomas (17y) and	[22]
							pancreatic tumor (29y) under chemotherapy.	This paper
XPElHaVI ^$^	North Africa	F		22	XP-C	delTG	Ovarian sarcoma (18y).	[22]
XPElKaVI ^$^	North Africa	F		28	XP-C	delTG	Uterine adenomyosarcoma (15y); death at 28.	[22]
XPMaAbVI	Morocco	M	22		XP-C	delTG	AML-3 (14y).	[22]
XPGaViVI *	Algeria	F		25	XP-C	delTG	Death of RAEB-2.	[22]
XPGaMVI *	Algeria	M		27	XP-C	delTG	Death of RAEB-t.	[22]
XPWaVI	Pakistan	M	33		XP-C	c.1243C>G; c.1934delC	Angiosarcoma of the inner canthus of the left eye (22).	[23]
XPMYVI	Comoros	F		30	XP-C	IVS 12-1G>C	Death of breast tumor at 30y.	[21]

HSCT: Hematopoeitic stem cell transplant; RAEB: Refractory Anemia with Excess Blasts. (*) Cousins; (**) Twins; (^$^) Sisters. delTG means c.1643_1644 delTG; p.Val548AlafsX572 *XPC* mutation.

**Table 2:** Different types of internal tumors according to the complementation groups in the French XP cohort.

Tumor Types *	Nb of Tumors **	Ages at Diagnosis	Ages at Death
		(Median Age in Years)	(Median Age in Years)
Hematological malignancies	23 (1 XP-D; 1 XP-V)	7–42 (25y)	10–42 (25y)
Gynecology	8	11–19 (17y)	13–28 (22y)
Brain	3	8–19 (10y)	8–19 (10y)
Thyroid	3	13–17 (17y)	19
Gastric	1 (XP-V)	48	54
Prostate	1 (XP-V)	60	na ***
Pancreas	1	29	na
Breast	1	30	30
Kidney	1	23	25
Angiosarcoma	1	22	na

(*) Forty-three tumors are reported for 40 XP patients because three XPs had two unrelated tumors (one XP-C had thyroid and pancreatic tumors, one XP-C had a gynecological tumor and AML, and one XP-C patient had two unrelated gynecological tumors). (**) All tumors were found in XP-C patients except the 4 ones indicated (3 XP-V and 1 XP-D). (***) na: means non-applicable.

## Data Availability

The data presented in this study are available in this article (and Supplementary Materials).

## Supplementary Materials

The following supporting information can be downloaded at: https://www.mdpi.com/article/10.3390/cancers15102706/s1, Table S1: Clinical and genetic description of the 181 XP patients of the French cohort.
