# Supplementary material for: The French Cohort of DNA Repair-Deficient Xeroderma Pigmentosum Patients: Risk of Hematological Malignancies
Source: Cancers (Basel). 2023 May 10;15(10):2706. doi: 10.3390/cancers15102706 (PMC10216379; doi:10.3390/cancers15102706)
Supplement: Supplementary file 1 [file cancers-15-02706-s001.zip › cancers-2362746-supplementary.pdf]

Supplementary Materials

The French Cohort of DNA Repair-Deficient Xeroderma Pigmentosum Patients: Risk of Hematological Malignancies

Alain Sarasin, PhD

UMR9019 CNRS, Gustave Roussy institute,
Villejuif, France
alain.sarasin@gustaveroussy.fr

Table S1: Clinical and genetic description of the 181 patients of the French cohort

Patient's code	Countries of family's origin	Sex	Ages in 2023 (years)	Ages at last seen (years)	Ages at death (years)	XP group	XP gene mutations	Clinical information
XP10VI*	Morocco	M			28	XP-C	delTG	AML-4 (27y); Death after chemotherapy at 28.
XP11VI*	Morocco	M			25	XP-C	delTG	Death of malignant melanoma.
XP14VI	French	M			13	XP-D		XP-D/Cockayne Syndrome (CS)
XP22VI	North Africa	F		18		XP-C	delTG	
XP31VI*	Algeria	F		26		XP-C		
XP32VI*	Algeria	F		18		XP-C		

XP34VI**	Gabon	M		31		XP-C		
XP36VI**	Gabon	M		33		XP-C		
XP51VI	Algeria	M		46		XP-V	c.1075_1244del; c.1091_1092insC	
XP56VI	Tunisia	F			52	XP-V	c.1075_1244del; c.1091_1092insC	
XP62VI	Algeria	M		33		XP-V	c.1075_1244del	
XP70VI	Tunisia	M		38		XP-C		
XP71VI	Algeria	M			14	XP-C	delTG	Death of malignant melanoma.
XP75VI	Algeria	M		43		XP-V	c.1091_1092insC	
XP78VI	Morocco	M		12		XP-C		
XP81VI*	Tunisia	M			24	XP-C	delTG	Death of malignant melanoma.
XP82VI*	Tunisia	M			18	XP-C	delTG	AML-6 (16y); HSCT (17y); Death of toxicity.
XP86VI	Algeria	M		53		XP-V	c.1561C>T	
XP90VI	Tunisia	F		24		XP-C	delTG	
XP92VI	North Africa	M		27		XP-C		
XPAlHaVI	Tunisia	M			25	XP-C	delTG	Death of AML.
XP93VI*	Tunisia	M	44			XP-C	del TG	
XP251VI*	Tunisia	M	32			XP-C	delTG	
XP252VI*	Tunisia	M	31			XP-C	delTG	
XP107VI	North Africa	F			17	XP-C		
XP114VI	Algeria	F		19		XP-C		
XP115VI	North Africa	F			25	XP-C		Death of malignant melanoma.
XP119VI	Algeria	F			21	XP-C		
XP127VI	French	M			57	XP-V	c.1093dupC	
XP128VI	North Africa	F			16	XP-C	delTG	Leukemia (15y); Death following chemotherapy.
XP131VI	French	M			47	XP-D		
XP134VI	North Africa	F		18		XP-C		
XP146VI	French	F		20		XP-V		
XP148VI	Algeria	F			19	XP-C	del TG	Poorly-differentiated follicular thyroid carcinoma (13y).
XP155VI	USA	M			42	XP-V		Death of T-lymphoma following chemotherapy.
XP159VI	French	F	44			XP-E	c.550G>C	
XP162VI	Mauritania	M		17		XP-A		XPA/CS syndrome.
XP163VI	Tunisia	F		22		XP-C		
XP165VI	Morocco	F			25	XP-C	delTG	Kidney adenocarcinoma (23y).
XP166VI	North Africa	F			31	XP-C		

XP167VI	Algeria	M			26	XP-C	delTG	Trisomy 21; MDS (25y); Death of AML.
XP185VI	Spain	F			25	XP-C	delTG	MDS (24y); Death of AML.
XP188VI*	Algeria	F		25		XP-C	delTG	
XP189VI*	Algeria	M		22		XP-C	delTG	
XP204VI**	French	M			29	XP-D	c.2125C>T	Death of neurological disorders
XP205VI**	French	M			32	XP-D	c.2125C>T	Death of neurological disorders
XP208VI	Algeria	M			17	XP-C	delTG	Mediastinal lymphoma (8y).
XP233VI*	Tunisia	M			8	XP-C	delTG	Death of anaplastic astrocytoma.
XP241VI*	Tunisia	F	30			XP-C	delTG	
XP433VI*	Tunisia	M	25			XP-C	delTG	
XP235VI	Tunisia	F			29	XP-C	delTG	MDS (24); Death of AML-6.
XP250VI	North Africa	F	32			XP-C		
XP256VI	French	F		48		XP-V	c.907C>T; c.1222_1225delACTT	
XP261VI*	French	F		31		XP-D	c.2125C>T; c.1925G>C	
XP266VI*	French	F		27		XP-D	c.2125C>T; c.1925G>C	
XP268VI	Greece	F	34			XP-C	c.779+1_14del12	
XP269VI	Morocco	F			23	XP-C	delTG	Cervical tumor (18y).
XP302VI	French	M		20		XP-C		
XP309VI	Morocco	F			10	XP-C	delTG	B-ALL (7y); Death of MDS following chemotherapy.
XP314VI***	French	M	30			XP-C	c.1243C>T; c.1704T>A	
XP315VI***	French	M	30			XP-C	c.1243C>T; c.1704T>A	
XP330VI	French	M	50			XP-C		
XP373VI	Algeria	F		14		XP-C	delTG	
XP401VI	French	M	27			XP-A		
XP415VI	Tunisia	M		29		XP-D	c.1802G>T	
XP420VI*	Morocco	F			25	XP-C	delTG	Death of cutaneous lymphoma NK.
XP423VI*	Morocco	F		36		XP-C	delTG	
XP422VI	French	F	74			XP-V	c.907C>T; c.1222_1225delACTT	
XP424VI	North Africa	M	43			XP-V		
XP435VI*	Algeria	M		9		XP-A	c.682C>T	
XP436VI*	Algeria	F		18		XP-A	c.682C>T	
XP440VI	Brazil	F	34			XP-G	c.83C>A; c.2904G>C	
XP442VI	North Africa	F		30		XP-D	c.1802G>T	
XP456VI*	North Africa	F	47			XP-A	IVS4 C.609-2 A>G	

XP457VI*	North Africa	M	40			XP-A	IVS4 C.609-2 A>G	
XP463VI	Algeria	F			9	XP-C		
XP475VI*	Algeria	M		23		XP-C		
XP476VI*	Algeria	F		20		XP-C		
XP480VI	Morocco	F	29			XP-C	delTG	
XP486VI	French	F	72			XP-V	c.907C>T	
XP491VI	Tunisia	M		78		XP-D	c.1880G>T	
XP492VI	Algeria	M		59		XP-D	c.1802G>T	
XP538VI	Algeria	M			29	XP-C	delTG	Death of AML-2.
XP546VI	Tunisia	M		37		XP-V	c.1091_1092insC	
XP551VI	French	F		35		XP-C	c.2089_2090insTGG	
XP552VI*	French	M			39	XP-D	R683W + del Ex 3	
XP553VI*	French	M			36	XP-D	R683W + del Ex 3	
XP574VI	North Africa	M		31		XP-V		
XP603VI	French	F	75			XP-V	c.1727_1728delCT; c.883G>A	
XP606VI*	French	F	53			XP-V	c.1727_1728delCT; c.883G>A	
XP737VI*	French	F	57			XP-V	c.1727_1728delCT; c.883G>A	
XP612VI	Algeria	F	24			XP-C	delTG	
XP627VI	French	M		42		XP-D	L461V + del 716-730	
XP629VI	North Africa	M	26			XP-C	delTG	Thyroid nodules
XP664VI*	Tunisia	M			10	XP-C	delTG	Death of astrocytoma.
XP665VI*	Tunisia	F		8		XP-C	delTG	
XPAdSaVI	Morocco	M			19	XP-C	delTG	Cerebellar astrocytoma (14y).
XP673VI	Morocco	F			22	XP-C	delTG	Death of T-ALL.
XP689VI	Morocco	F	22			XP-A	IVS4 C.609-2 A>G	
XP694VI	North Africa	F	21			XP-C	delTG	Left Ovarian juvenile granulosa-cell tumor at 19y and right ovarian Sertoli-Leydig cell tumor at 19y.
XP695VI	French	F		82		XP-C	c.2108C>T; c.2544delA	The oldest XP-C patient in our cohort.
XP700VI*	Morocco	F		26		XP-A	c.682C>T	
XP701VI*	Morocco	F		20		XP-A	c.682C>T	
XP704VI	French	F	44			XP-C	c.1243C>T; c.1399C>T	
XP719VI*	Morocco	F			17	XP-A	IVS4 C.609-2 A>G	Death of fulminant hepatitis.
XP851VI*	Morocco	M	16			XP-A	IVS4 C.609-2 A>G	
XP731VI	North Africa	M	43			XP-C	delTG	

XP744VI	French	M	20			XP-C	c.463C>T; c.2287delC	
XP757VI	French	M			20	XP-D		Hodgkin Disease (20y). Death following chemotherapy.
XP769VI*	North Africa	M	23			XP-C	delTG	
XPKaIbVI*	North Africa	M	20			XP-C	delTG	
XP783VI	North Africa	M	21			XP-A	c.682C>T	
XP791VI	North Africa	M	19			XP-C	delTG	A germline mutation on *MEN1* gene.
XP798VI*	North Africa	F	19			XP-C	delTG	
XP855VI*	North Africa	M	17			XP-C	delTG	
AS802VI	Algeria	M	33			XP-C	delTG	Poorly-differentiated follicular thyroid carcinoma (20y).
XP819VI	French	M	73			XP-V	c.1727_1728delCT; c.883G>A	Prostate tumor at 60y.
XP820VI*	North Africa	F	19			XP-C	delTG	MDS/AML in January 2023 waiting for HSCT.
XP821VI*	North Africa	F	21			XP-C	delTG	
XP822VI	Spain	F	18			XP-D	c.1867_1868insG; c.2164C>T	Syndrome XPD/TTD.
XP826VI	North Africa	M	19			XP-C	delTG	
XP843VI	North Africa	M		22		XP-V	c.797T>A	
XP853VI	North Africa	M			36	XP-V	c.278G>C	
XP858VI	Morocco	F		13		XP-A	c.682C>T	
XP860VI	North Africa	F			29	XP-C	c.658C>T	Death of malignant melanoma of the face.
XP865VI	North Africa	M	32			XP-V		
XP871VI	Australia	F		9		XP-F	R589W + del Ex 3 31 nt	
XP872VI	Kosovo	F	40			XP-V	c.2074A>G; c.2141A>G	
XP874	Australia	M	16			XP-D	c.1484T>C; c.1775G>C	Syndrome XPD/TTD.
XP879VI	North Africa	M	27			XP-C	C.1103_1104delAA; delTG	
XP888VI*	North Africa	M	36			XP-V	c.672delA; IVS1+1G>C	
XP968VI*	North Africa	F	43			XP-V	c.672delA; IVS1+1G>C	
XP892VI	North Africa	M	18			XP-C	delTG	
XP893VI	North Africa	M	18			XP-C	delTG	
XP922VI	French	M	15			XP-C		
XP924VI*	Morocco	M			15	XP-C	delTG	T-ALL (12y); MDS (13y); Death of AML-6.
XPEsAdVI*	Morocco	M	18			XP-C	delTG	
XP934VI	French	F	14			XP-C	c.658C>T; c.1243C>T	
XP961VI	French	F	55			XP-V	c.437dupA	
XP964VI	French	F	53			XP-E		
XP965VI	North Africa	F	55			XP-V	c.907C>T	

XP966VI*	French	F		88	XP-V	c.108_110del3	
XP967VI*	French	F	74		XP-V	c.108_110del3	
XP2003VI***	Algeria	F	28		XP-C	delTG	Vaginal embryonal rhabdomyosarcoma at 16y.
XP2004VI***	Algeria	F		23	XP-C	delTG	Vaginal rhabdomyosarcoma at 16y and death of AML.
XP2006VI*	Morocco	F	34		XP-C	delTG	AML-6 (29y); HSCT (29y).
XPToMoVI*	Morocco	M	29		XP-C	delTG	
XPBoMoVI	North Africa	M	32		XP-C		
XPBoNaVI	North Africa	F	31		XP-C		
XPGAVI	French	F		54	XP-V	c.1727_1728delCT; c.883G>A	Gastric tumor (48y).
XPChFaVI	North Africa	M	28		XP-C	delTG	
XPAHVI	Tunisia	M		25	XP-C	delTG	AML (24y). Curietherapy (24y). Death at 25.
XPChFa2VI	North Africa	M		24	XP-C		Death of leukemia.
XPCoJoVI	French	F	29		XP-C		
XPMoAbVI	North Africa	M	35		XP-C		
XPBeDeVI	North Africa	F	11		XP-C		
XPBoIlVI	North Africa	M	17		XP-D	c.A254G	
XP2020VI	Tunisia	F	14		XP-C	delTG; c.G850T	Ovarian Sertoli-Leydig cell tumor (11y).
XPDuMeVI	North Africa	F	18		XP-C		
XPRaWaVI*	North Africa	M	10		XP-C	delTG	
XPRaYaVI*	North Africa	F	13		XP-C	delTG	
XPAAVI	Algeria	F	29		XP-C	delTG	Papillary thyroid carcinomas (17y) and pancreatic tumor (29y) under chemotherapy.
XPElHaVI*	North Africa	F		22	XP-C	delTG	Ovarian sarcoma (18y).
XPElKaVI*	North Africa	F		28	XP-C	delTG	Uterine adenomyosarcoma (15y). Death at 28.
XPMaAbVI	Morocco	M	22		XP-C	delTG	AML-3 (14y).
XPGaViVI*	Algeria	F		25	XP-C	delTG	Death of RAEB-2.
XPGaMVI*	Algeria	M		27	XP-C	delTG	Death of RAEB-t.
XP1BEVI	Belgium	M	27		XP-V		
XPWaVI	Pakistan	M	33		XP-C	c.1243C>G; c.1934delC	Angiosarcoma of the inner canthus of the left eye (22y).
XPAbLyVI	North Africa	F	28		XP-C		
XP844VI	Comoros	M	19		XP-C	IVS 12-1G>C	
XP845VI*	Comoros	M	27		XP-C	IVS 12-1G>C	
XPMYVI*	Comoros	F		30	XP-C	IVS 12-1G>C	Death of breast tumor at 30y.
XP936VI	Comoros	M	22		XP-C	IVS 12-1G>C	

XPYoIsVI	Comoros	M	25		XP-C	IVS 12-1G>C	
XPMa01VI	Comoros	F	31		XP-C	IVS 12-1G>C	
XPMa02VI	Comoros	M	24		XP-C	IVS 12-1G>C	
XPMa14VI	Comoros	M	22		XP-C	IVS 12-1G>C	
XPMa06VI	Comoros	F		32	XP-C	IVS 12-1G>C	Death of malignant melanoma.
XPMa09VI	Comoros	F	21		XP-C	IVS 12-1G>C	
XPBANaVI	Comoros	F	21		XP-C	IVS 12-1G>C	Trichoblastoma.

- (*) or (**): siblings of the closest XP patient in the list; (***): twins.
- HSCT: hematopoietic stem cell transplant; RAEB; refractory anemia with excess blasts.
- delTG means c.1643_1644 delTG; p.Val548AlafsX572 *XPC* mutation.
- CS means Cockayne Syndrome which is a DNA repair-deficient disease with neurological abnormalities and early aging but no increased risk of tumors.
- TTD means trichothiodystrophy which is a DNA repair-deficient disease with neurological abnormalities but no increased risk of tumors.
